# Counterillumination in a large demersal bioluminescent shark

**DOI:** 10.1016/j.isci.2026.117089

**Published:** 2026-08-06

**Authors:** Julien M. Claes, Darren W. Stevens, Jérôme Mallefet

**Affiliations:** 1 Marine Biology Laboratory, Earth and Life Institute, Université Catholique de Louvain, 1348 Louvain-la-Neuve, Belgium; 2 Zoovery SRL, 1640 Rhode-Saint-Genèse, Belgium; 3 Earth Sciences New Zealand, Private Bag Wellington 14901, New Zealand

**Keywords:** bioluminescence, shark, deep-sea, counterillumination, photophore, *Dalatias licha*, vision, camouflage

## Abstract

Counterillumination is a widespread camouflage strategy in mesopelagic organisms, whereby ventral light emission reduces visibility from below. While its predator-avoidance camouflage function is well established, its potential role in predation remains poorly supported. Here, we investigate bioluminescence in the kitefin shark (*Dalatias licha*), a large demersal predator for which no natural predators have been recorded. Using *in vivo* luminescence measurements, we show that this species produces a spatially uniform ventral emission—with no significant difference in radiance across body regions—whose intensity and spectral peak are consistent with a counterillumination function. However, its ecological context, combined with conspicuous dorsal luminescence and a broad angular emission pattern, instead points to a primary role in prey detection and capture rather than predator-avoidance camouflage. These findings expand the functional framework of counterillumination and highlight its ecological relevance in deep benthopelagic environments.

## Highlights

• Ventral bioluminescence in kitefin shark matches downwelling daylight
• Its angular light emission is far broader than other counterilluminators
• Demersal ecology and broad emission point to a role in prey capture
• Dorsal fin glow exceeds ambient light, suggesting a signaling function

## Graphical abstract

**Figure:**
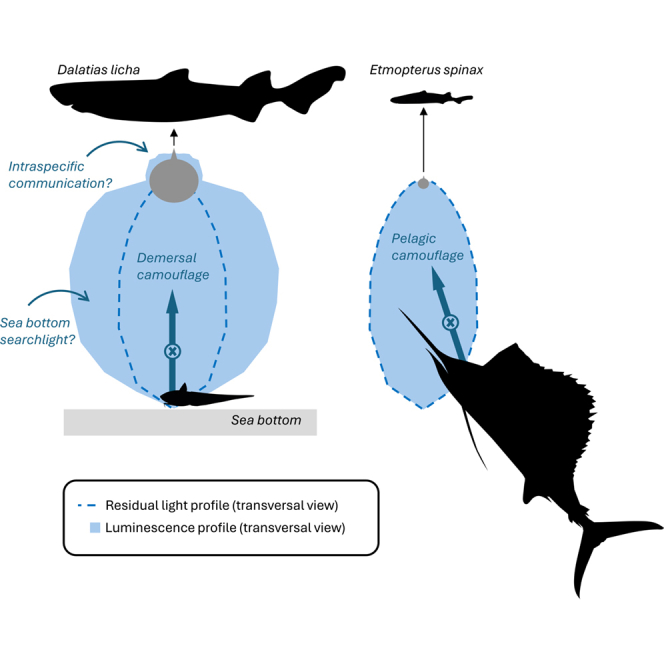

## Teaser

Biological sciences; Zoology; Animals

## Introduction

Counterillumination is one of the most widespread camouflage strategies in the pelagic realm, whereby animals emit ventral light to erase their silhouette when viewed from below.^1^^,^^2^^,^^3^ It is a common cryptic adaptation among fishes, squids, and crustaceans inhabiting the mesopelagic zone,^4^^,^^5^^,^^6^^,^^7^^,^^8^^,^^9^^,^^10^ where residual downwelling light creates strong visual contrast. These organisms possess ventrally oriented photogenic organs (photophores), often coupled with sophisticated optical structures—such as lenses, filters, and reflectors—and regulatory feedback mechanisms that enable precise matching of the intensity, spectrum, and angular distribution of ambient light, thereby ensuring effective concealment.^11^^,^^12^^,^^13^^,^^14^^,^^15^^,^^16^^,^^17^^,^^18^^,^^19^

The antipredatory value of counterillumination has been experimentally demonstrated—for example in the midshipman fish (*Porichthys notatus*)^20^ and in a field experiment using illuminated seal decoys attacked by white sharks^21^— and is widely considered its primary function. This interpretation is consistent with the ecology of most counterilluminating organisms, which are typically small, exposed, and highly vulnerable in the water column. In contrast, convincing evidence that counterillumination may reduce detection of the predator by prey has remained elusive, and the functional scope of this strategy is therefore likely underestimated.

The kitefin shark (*Dalatias licha*) provides a unique opportunity to revisit this paradigm. As the largest known bioluminescent vertebrate, reaching up to 1.8 m in length,^22^ it differs fundamentally from typical counterilluminators. Its skin contains millions of photophores, most densely distributed not only on the ventral surface—where they can reach several thousand per cm^2^—but also extending along the flanks, dorsal trunk, and dorsal fins, producing a continuous whole-body glow.^23^ This predominantly ventral luminescence has led to the hypothesis that the species employs counterillumination, yet this interpretation has never been tested in its ecological context. At the same time, this photophore pattern gives rise to a 2-fold ecological paradox. First, *D. licha* is a predominantly demersal species for which no natural predators have been recorded, making a predator-avoidance camouflage role for counterillumination unlikely. Second, the presence of both ventral and dorsal luminescence departs from the classical counterillumination pattern, in which emission is restricted to the ventral surface.^24^

Here, we characterize bioluminescence in live kitefin sharks freshly captured by bottom trawl and test the counterillumination hypothesis by comparing the physical properties of their luminescence to predicted ambient light conditions at capture depth. We first measured the spectral peak and ventral radiance of *D. licha* and compared these to the predicted downwelling light field at depth, then quantified the angular distribution of ventral emission. We subsequently compared the angular emission profile of *D. licha* to that of all counterilluminating organisms for which such data are available, including lanternsharks (*Etmopterus* spp.) and the bioluminescent dalatiid shark *S*. *aliae*, as established reference counterilluminators. Given that *D. licha*, like all bioluminescent sharks, possesses a high density of small, pigmented, outward-facing photophores^23^^,^^25^^,^^26^^,^^27^^,^^28^ that collectively allow the ventral surface to be approximated as a homogeneous photogenic emitting layer,^24^ ventral radiance was derived from measured photon flux under a Lambertian approximation. We show that *D. licha*—a large, slow-swimming demersal predator for which no natural predators have been recorded—produces ventral emission consistent with counterillumination, with an angular emission profile markedly broader than that of all other counterilluminating taxa for which such data are available, while also displaying dorsal fin luminescence that, although weaker than ventral emission, substantially exceeds ambient upwelling radiance. This combination supports the interpretation that counterillumination in this species primarily contributes to prey detection and capture rather than predator-avoidance camouflage.

## Results

### Ventral luminescence is spatially uniform and consistent with counterillumination

**Figure 1:**
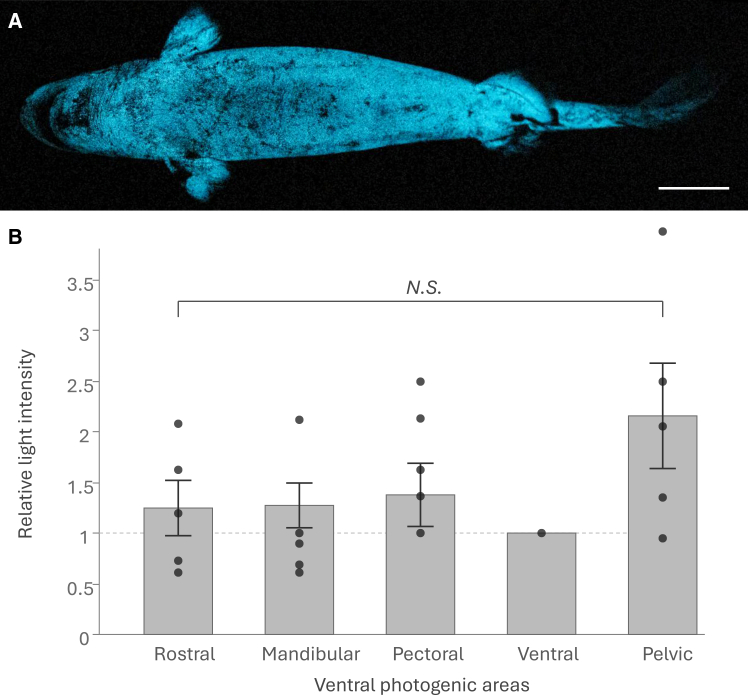
Ventral spontaneous luminescence in the kitefin shark *Dalatias licha* (A) Spontaneous ventral bioluminescence in a live *D. licha*. (credit: J. Mallefet). Scale bars, 10 cm. (B) Relative luminescence intensity of ventral photogenic areas. Luminescence intensity was measured at 1 cm from the surface of each photogenic area and normalized to the intensity of the ventral surface of the same individual. Bars represent mean relative intensity ±standard error of the mean (*n* = 5); dots show individual values. The dashed line indicates the reference value of 1 (ventral surface). No significant difference was detected among areas (N.S.).

During the survey, 24 *D. licha* were obtained as bycatch, of which eight were alive and displayed spontaneous, stable bioluminescence upon placement in darkened tanks; these individuals were selected for analysis (Table S1). Ventral luminescence peaked in the blue-green region (491 nm) and its intensity did not differ significantly among anatomical zones of the ventral surface (repeated-measures ANOVA, F_8_,16 = 2.69, *p* = 0.069; Friedman, χ^2^(4) = 6.08, *p* = 0.193), indicating that the ventral photogenic side—representing >95% of the projected silhouette including fins—acts as a homogeneous light source (Figure 1). Ventral shark radiance (21.9–53.5 Tq m^−2^ s^−1^ sr^−1^) matched predicted downwelling daylight at depths of 480–540 m, which fall within the observed capture depth range and do not differ significantly from median capture depths with both parametric (paired Student’s *t* test, t(7) = −1.40, *p* = 0.204) and non-parametric approaches (Wilcoxon signed-rank test, W = 11, *p* = 0.383).

### Angular emission is markedly broader than predicted for classical counterillumination

**Figure 2:**
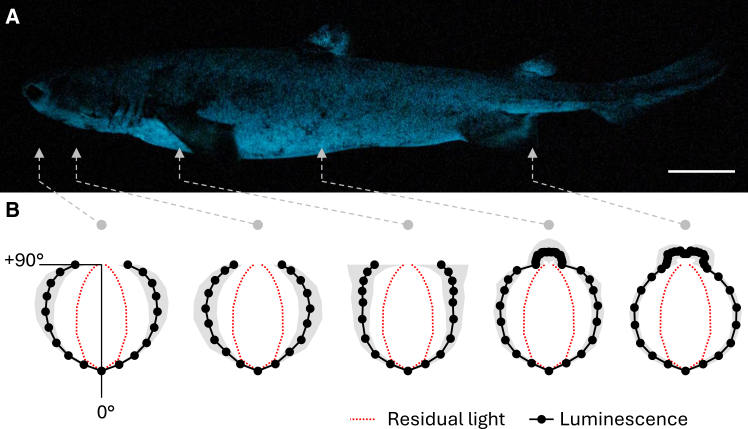
Angular distribution of luminescence in the kitefin shark *Dalatias licha* (A) Spontaneous lateral luminescence in a live *D. licha*. (credit: J. Mallefet). Scale bars, 10 cm. (B) Transverse angular emission at five body positions (white arrows, from left to right: rostral, mandibular, pectoral, ventral and pelvic). Polar profiles are constructed using mean relative luminescence intensity measured around the shark body with 10° increment (light gray shading indicates standard error); while rostral, mandibular and pectoral profiles only show ventral output (downward hemisphere), ventral and pelvic profiles combine both ventral and dorsal output to include contributions from the first and second dorsal fins, respectively; dotted red curves represent the predicted angular distribution of downwelling light (theoretical Tyler distribution^11^). Scale bars in (A), 10 cm.

**Figure 3:**
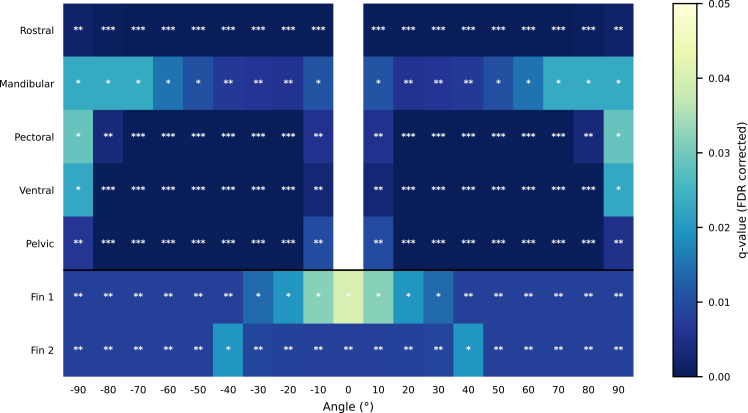
Comparison of measured angular emission distributions with the theoretical Tyler distribution across all anatomical regions and fins Heatmap of *q* values (FDR-corrected *p* values, Benjamini-Hochberg) from two-sided one-sample Student’s *t* tests comparing, for each angle, observed values with the value expected from the Tyler model (relative distribution normalized at 0°). Ventral photogenic areas (rostral, mandibular, pectoral, ventral, pelvic) and dorsal fins (fin 1, fin 2) are shown together and separated by a horizontal line. For dorsal fins, Tyler-expected values were divided by 200 to represent the “dorsal shield,” without modifying observed values. Angles are displayed symmetrically (−θ and +θ). The 0° angle (normalization point) was excluded from statistical tests. The color scale (light green to blue) represents corrected *q* values and is saturated at 0.05 to improve readability. Asterisks ∗, ∗∗, and ∗∗∗ indicate *q* < 0.05, *q* < 0.01, and *q* < 0.001, respectively.

**Figure 4:**
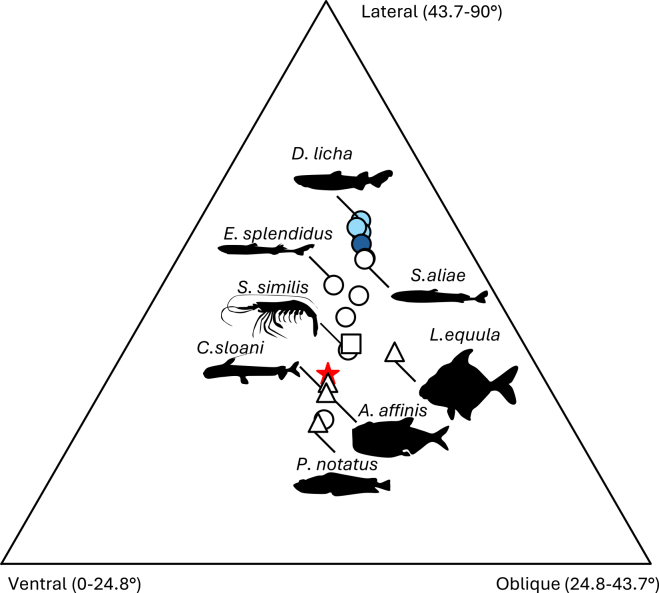
Angular distribution of light in counterilluminating animals Comparative angular emission of counterilluminating animals plotted in ternary space relative to downwelling light (red star), based on published and unpublished angular emission data, showing a pronounced lateral deviation of *D. licha* from other taxa (see supplemental information). Blue circles indicate transverse angular emission profiles of *D. licha* (light blue indicate anterior anatomical areas, from top to bottom: rostral, pectoral, mandibular; dark blue indicate posterior anatomical areas, from top to bottom: ventral, pelvic); open circles, sharks; open triangles, bony fishes; open square, crustacean (animal outlines: J.M. Claes).

The angular radiance profile of *D. licha*, measured at five body regions from −90° to +90° relative to vertical, exhibited a significantly broader angular distribution than downwelling daylight (Figure 2), with radiance exceeding Tyler’s model prediction at all measured angles (FDR-corrected *p* < 0.05; Figure 3; Tables S2 and S3). This pattern differs from all other counterilluminating animals for which angular data are available, including luminous sharks (permutation test, *p* = 0.02; Figure 4). This was even more pronounced in the anterior regions—e.g., rostral, mandibular, and pectoral—whose emission was broader than that of posterior regions, e.g., ventral and pelvic (permutation test, *p* = 0.02; Figure 4). In contrast, *Etmopterus* spp. emission did not differ significantly from the predicted downwelling field (permutation test, *p* = 0.21), indicating close angular matching.

### Dorsal fin luminescence exceeds ambient upwelling light

Transverse measurements further revealed dorsal fin luminescence in *D. licha* that, while weaker (by approximately one order of magnitude; Figure 3) in absolute radiance than ventral surface, exceeded exceeding ambient upwelling light across all viewing angles by approximately two orders of magnitude, rendering the dorsal fins conspicuous against the background light field (FDR-corrected *p* < 0.05; Figure 2).

## Discussion

Our measurements demonstrate that *D. licha* produces a spatially uniform ventral emission whose intensity and spectral peak are consistent with the physical requirements of counterillumination: ventral radiance matches the downwelling light field at the observed capture depths, and the emission maximum (491 nm) corresponds closely to the spectral peak of downwelling irradiance at mesopelagic depths (472–489 nm^22^^,^^29^). The ecological function served by this matching—and therefore the selective pressure that maintains it—is an inference we develop in the following text, drawing on the ecology, morphology, and behavior of the species. Notably, although sharks were brought from depth to the surface during capture and thus exposed to isolumes far exceeding those at their capture depth, the ventral radiance measured at the surface on freshly captured individuals was not significantly different from the downwelling irradiance modeled at their capture depth. This suggests that *D. licha* may operate as an isophotic counterilluminator, a pattern previously described in other bioluminescent sharks and interpreted as a physiological constraint imposed by the hormonal regulation of their photophore output.^25^ Indeed, bioluminescent sharks generally show limited capacity for active, graded intensity control^30^: photophores contain few photocytes—a single cell in Dalatiidae, around a dozen in Etmopteridae^23^^,^^26^^,^^27^^,^^28^—and hormonal control does not target individual cells, precluding the progressive photocyte recruitment that would otherwise allow pronounced intensity modulation. Telemetry data further indicate that *D. licha* undergoes comparatively restricted vertical movements (typically less than 150 m difference between day and night depths) and remains closely associated with the seafloor,^31^ consistent with an isophotic counterillumination strategy that does not require coping with large fluctuations in ambient light.

*D. licha* exhibits the longest peak emission wavelengths reported among bioluminescent sharks. In contrast, other dalatiid sharks peak in the dark blue range (e.g., 455 nm for *Isistius brasiliensis*,^32^ 457 nm for *S*. *aliae*^25^). Such greener luminescence is commonly observed in seafloor-associated organisms^33^^,^^34^ and may reflect the optical properties of near-bottom waters: suspended sediments containing chlorophyll-derived particles could shift light transmission toward longer wavelengths relative to open pelagic environments,^34^ thereby improving spectral matching with the ambient downwelling field. Although the spectral composition of downwelling light near the Chatham Rise seafloor remains uncharacterized, this hypothesis is testable through *in situ* optical measurements.

Bycatch sharks may undergo a disturbed physiological state, so we restricted our analyses to the eight individuals that showed spontaneous, stable luminescence throughout the observation period. This choice reflects both methodological and physiological considerations: reliable angular and intensity measurements require a luminescent signal that can be sampled consistently over several minutes, and in our experience, luminescence also fades before death in bioluminescent sharks—including the dalatiid *S*. *aliae* and the etmopterids *Etmopterus* *schmidti*, *E*. *spinax* and *E. splendidus*—while counterilluminating animals more generally maintain a stable output at a given level of illumination.^5^ By working only with stable-output individuals, we minimized the risk of measuring compromised animals.

The central finding of this study, however, concerns not intensity or spectrum but angular emission. For a mesopelagic organism seeking to hide from predators attacking from below, effective counterillumination requires matching the downwelling light field at every angular direction from which a predator could view it—a stringent, omnidirectional requirement spanning the entire downward hemisphere. This is precisely what is observed in pelagic counterilluminators: their angular emission profiles closely track the theoretical Tyler distribution^11^ across all measured angles, as confirmed here for *Etmopterus* spp.

The ecology of *D. licha*, however, differs fundamentally from that of pelagic counterilluminators. As a large demersal predator hunting prey located close to the seafloor below it, the relevant visual observers are not predators looking upward from open water but prey looking upward at a narrow range of near-vertical angles. To suppress its own silhouette against the downwelling light field as seen by prey directly below, *D. licha* need only match that field within a restricted near-vertical angular window. The constraint on lateral emission angles is therefore fundamentally relaxed: it is only at large lateral distances that the shark's emission would be detectable against the downwelling light field—and at such distances, prey are outside the attack envelope of a predator that hunts downward using a ventral mouth and could not pursue them in any case.

Consistent with this relaxed lateral constraint, the angular emission profile of *D. licha*, measured in the transverse plane, is markedly broader than the Tyler distribution at all measured angles and differs significantly from all other counterilluminating animals for which angular data are available, including other bioluminescent sharks. This broadening is most pronounced in the anterior regions—rostral, mandibular, and pectoral—whose emission is significantly broader than that of posterior regions. One parsimonious explanation is that this excess lateral emission functions as a biological searchlight, illuminating the seafloor to the sides of the shark and potentially enhancing prey detection in the otherwise dark benthic environment. The use of bioluminescence as a searchlight for prey detection is well established in other taxa: the splitfin flashlight fish (*Anomalops katoptron*) actively increases light-organ open time during prey capture, with luminescent individuals achieving ∼7× higher catch rates than non-luminescent conspecifics,^35^ and several deep-sea stomiiform fishes (e.g., *Malacosteus* spp., *Aristostomias* spp.) produce far-red bioluminescence matched to their own visual pigments but invisible to most prey, functioning as a private searchlight channel.^36^^,^^37^^,^^38^ Consistent with the searchlight hypothesis, many deep-sea animals have independently evolved extremely low body reflectance that reduces their visibility to biological searchlights,^39^ suggesting strong selection pressure from bioluminescent predators using their own light to detect prey.

The lateral geometry of the emission broadening is further supported by the visual morphology of *D. licha*. The large, laterally positioned eyes of this species maximize the lateral visual field—a configuration adapted for detecting objects in the lateral hemisphere rather than directly below the snout. Notably, the other taxa invoked as evidence for the biological searchlight strategy share this geometry: the sub-ocular light organ of *A*. *katoptron* illuminates the lateral visual field in the direction of the fish’s lateral visual axis,^35^ and the far-red photophores of *Malacosteus* spp. and *Aristostomias* spp. are similarly positioned to illuminate the lateral visual fields of these laterally eyed stomiiform fishes.^40^ This convergence of lateral bioluminescence and lateral visual orientation across phylogenetically distant taxa strengthens the case for a lateral searchlight function in *D. licha*.

A second, complementary consideration further relaxes the requirement for direct ventral illumination at the moment of prey capture. Once prey has been detected and approached, *D. licha* may not need to maintain visual contact with prey positioned immediately below its snout to complete the strike: elasmobranchs possess the ampullae of Lorenzini, a short-range electroreceptory system that detects the bioelectric fields produced by all living organisms,^41^ which provide close-range prey guidance independently of vision. We note, however, that direct demonstration of the searchlight function—and of any sequential use of visual and electrosensory systems during prey capture—would require *in situ* behavioral observations; the data presented here are consistent with this hypothesis but do not confirm it.

Far from being contradictory, the intensity and spectral matching (consistent with counterillumination) and the lateral angular broadening (inconsistent with classical pelagic counterillumination) are therefore jointly explained by a single functional framework: *D. licha* simultaneously suppresses its near-vertical silhouette as seen by prey below, and deploys laterally broad luminescence to illuminate the prey field—two functions that are ecologically compatible for a large demersal predator hunting on the seafloor but would be mutually exclusive for a vulnerable counterilluminator in open water.

Multiple independent lines of evidence support this functional interpretation, though behavioral experiments would be required to demonstrate it directly. Tracking data show that *D. licha* remains tightly associated with the seafloor,^31^ making encounters with predators attacking from below unlikely and rendering a predator-avoidance function for counterillumination implausible. Its diet includes fast-moving, visually acute prey such as cephalopods and lanternsharks^42^^,^^43^^,^^44^—taxa with sensitive visual systems^45^^,^^46^ and rapid escape responses^47^^,^^48^—and its attack strategy from above using a ventral mouth makes visual stealth during approach a plausible and likely critical selective pressure. This is reinforced by the fact that *D. licha* is the slowest-swimming shark on record (∼0.13 m s^−1^),^47^ making a surprise-based attack strategy likely of high adaptive value. The large lateral eyes of *D. licha*, which have the highest pupil-to-body ratio reported in Dalatiidae,^22^ are consistent with high sensitivity to the dim bioluminescent field that would be generated by its own ventral emission near the seafloor. We note that this prey-detection interpretation does not require any mechanistic reinterpretation of counterillumination—the optical principle is identical whether the relevant observer is a predator or prey. What differs is the selective context: the identity of the visual observer whose detection the organism is evolving to avoid.

The few prior hypotheses proposing a predatory use of counterillumination have been restricted to bioluminescent sharks and have remained conceptually contested. The cookiecutter shark (*Isistius brasiliensis*) has been proposed to use its dark ventral collar as a lure while counterillumination conceals the rest of its body,^49^ a hypothesis that remains mechanistically problematic as luring requires enhancing rather than suppressing detectability.^25^ The velvet belly lanternshark (*Etmopterus spinax*) has been suggested to approach prey undetected using counterillumination—a hypothesis first proposed on qualitative grounds by Hickling^50^ and subsequently supported by live luminescence data recording^30^—but this species remains small (maximum 60 cm^22^), partially pelagic (as indicated by its diet dominated by pelagic prey^51^^,^^52^^,^^53^), and itself subject to predation (including by *D. licha*^42^), making a primarily predator-avoidance role more parsimonious. The combination of ecological traits unique to the kitefin shark—its trophic position, demersal lifestyle, extreme slowness, dependence on visually acute prey, and markedly broader lateral emission profile relative to all known pelagic counterilluminators—makes this species the most compelling case to date for a counterillumination system primarily shaped by prey capture rather than predator-avoidance selective pressures.

The dorsal fin luminescence, while weaker in absolute radiance than the ventral surface emission, exceeds ambient upwelling radiance by approximately two orders of magnitude at all measured angles, rendering the dorsal fins conspicuous when viewed laterally against the background light field. This pattern is incompatible with any camouflage function and is more consistent with intraspecific signaling or prey attraction.^9^^,^^54^ Given the anatomical separation from the mouth and recent evidence of aggregative behavior in *D. licha*,^31^^,^^55^ intraspecific communication appears the more plausible role, though *in situ* observations will be required to discriminate between these possibilities.

Collectively, our findings illustrate how counterillumination can subserve opposite ecological functions depending on the identity of the relevant visual observer—a striking example of how perceptual design, the evolutionary shaping of bioluminescent traits by the sensory and ecological properties of the receiver,^54^ operates independently of the underlying physics of emission. The relaxation of the lateral angular constraint in *D. licha*, and its apparent co-option as a lateral illumination field, illustrate how the ecology of the observer, not merely the physics of the emission, determines the selective landscape of bioluminescence. These results extend the functional scope of counterillumination to benthopelagic deep-sea ecosystems and underscore how much of the ecological logic of the deep sea—the Earth’s largest and most uncharted biome—remains to be deciphered.

### Limitations of the study

This study has several limitations. First, our analyses are based on eight bycaught individuals from a single survey and season; broader sampling across sex, size classes, depths, and seasons would strengthen the generality of our findings. Second, we cannot determine why luminescence was absent or declining in the bycatch individuals not retained for analysis: this could reflect capture and emersion stress, an attempt to adjust to the markedly different light regime experienced during ascent, or some other cause. Sharks were captured across a wide window (06:00–24:00 h) spanning a broad range of ambient light conditions, and we did not record the time elapsed since each individual was brought to the surface, which would be needed to investigate this further. We addressed this uncertainty conservatively, by restricting our measurements to the eight individuals with a stable luminescent output, rather than by attempting to adjudicate between these explanations. Third, our estimate of counterillumination depth relies on a Lambertian approximation of ventral emission and on a generic oceanic downwelling-light model that has not been directly validated against *in situ* measurements near the Chatham Rise seafloor; the spectral composition of near-bottom downwelling light in this habitat also remains uncharacterized. Fourth, replication was limited for some comparative taxa (e.g., *Etmopterus* spp.), constraining the statistical power of between-taxon comparisons. Finally, our interpretation of the functional significance of dorsal fin luminescence, and of the proposed lateral searchlight role of ventral emission in prey detection, rests on indirect ecological and morphological evidence; direct behavioral confirmation, ideally through *in situ* observation of free-swimming *D. licha*, will be required to test these hypotheses directly.

## Resource availability

### Lead contact

Further information and requests for resources and reagents should be directed to and will be fulfilled by the lead contact, Julien M. Claes (julien.claes@gmail.com).

### Materials availability

This study did not generate new unique reagents. Voucher specimens of *D*. *licha* generated during this study are kept at Marine Biology Laboratory (Earth and Life Institute, Université catholique de Louvain, Belgium) and are available from the lead contact upon request.

### Data and code availability

• All luminescence intensity data generated in this study have been provided as tables in the supplemental information or a separate Excel file (Table S2).
• This study did not produce new code.
• Any additional information required to reanalyze the data reported in this paper will be shared by the lead contact upon request.

## Acknowledgments

The authors acknowledge Richard O’Driscoll, Program Leader—Fisheries Monitoring Earth Sciences New Zealand, the scientific staff, and the skillful crew of R.V. Tangaroa on voyage TAN2401 (Chatham Rise trawl survey) and Fisheries New Zealand for funding the survey under project MID2021-02.). J.M.C. is a Scientific Collaborator of the Université catholique de Louvain (Marine Biology Laboratory). J.M. is Research Associate to F.R.S.–FNRS. This paper is a contribution to the Earth and Life Institute-Biodiversity Research Center BRC # 445 (ELIV) and the Centre Interuniversitaire de Biologie Marine (CIBIM). We thank four reviewers for exceptionally detailed and constructive engagement with this manuscript; their comments prompted important clarifications in the functional interpretation, methodological justification, and presentation of the data.

## Author contributions

J.M. and D.W.S. carried out field work including spontaneous luminescence data recordings and pictures. J.M.C curated the dataset and conducted the analyses. J.M.C. wrote the initial draft of the manuscript and designed the figures including the graphical abstract, with subsequent input from all the other authors.

## Declaration of interests

The authors declare no competing interests.

## Declaration of generative AI and AI-assisted technologies in the writing process

During the preparation of this manuscript, ChatGPT (OpenAI; GPT-5.) and Claude (Anthropic) were used solely for language polishing and grammar checking of the manuscript. All output text was critically reviewed and edited, and the authors take full responsibility for the final content.

## STAR★Methods

### Key resources table

**Table:** 

REAGENT or RESOURCE	SOURCE	IDENTIFIER
**Deposited data**		

Luminescence intensity data for *D. licha* and comparative taxa	This paper	See supplemental information

**Experimental models: Organisms/strains**		

Kitefin shark (*D. licha*), wild-caught bycatch, Chatham Rise, New Zealand	R.V. Tangaroa survey TAN2401, Earth Sciences New Zealand	N/A

**Software and algorithms**		

Python 3 (SciPy, statsmodels)	Python Software Foundation; SciPy community	https://www.python.org; https://scipy.org
Fiji/ImageJ (v2.14.0/1.54f)	National Institutes of Health	RRID:SCR_002285
MagicPlot	MagicPlot Systems	https://magicplot.com
Adobe Photoshop	Adobe Inc.	https://www.adobe.com
Microsoft PowerPoint (Microsoft Office suite)	Microsoft Corporation	https://www.microsoft.com

**Other**		

Sony α7S III digital camera	Sony Corporation	Model ILCE-7SM3
Hamamatsu mini spectrometer	Hamamatsu Photonics	C10083CA
Optical fiber (spectrometer)	Hamamatsu Photonics	A976201
Optical fiber probe (luminometer)	Edmund Optics	#39-368
Photon-counting luminometer	Berthold Technologies	FB12
Beta light calibration source (470 nm)	Saunders Technology	N/A

### Experimental model and study participant details

#### Shark collection

Kitefin sharks (*Dalatias licha*) were obtained as fisheries bycatch during a hoki stock assessment survey conducted aboard R.V. *Tangaroa* by Earth Sciences New Zealand (formerly the National Institute of Water and Atmospheric Research, abbreviated as NIWA) for the New Zealand Ministry for Primary Industries on the Chatham Rise in January 2024, at depths of 489–653 m.^56^ Sharks were temporarily maintained in darkened tanks supplied with fresh, cold seawater prior to observation, and each individual was photographed in complete darkness. All specimens were wild-caught, free-ranging *D. licha* of undetermined genetic strain (a non-model, wild elasmobranch species); sex and total length were recorded for each of the eight individuals retained for analysis and are reported in Table S1 (7 males, 1 female; total length range 39–85 cm). Individuals ranged from juvenile to adult based on size and morphology. Sample size was insufficient to formally test for an effect of sex on luminescence intensity or angular emission; this is acknowledged as a limitation of the study (see limitations of the study). This study did not involve established cell lines, primary cell cultures, or human participants.

All animal handling followed Earth Sciences New Zealand's Code of Ethical Conduct.^57^ Observations were non-invasive; angular emission measurements involved brief emersion of individuals placed in dorsal recumbency (tonic immobility) for ∼2–3 min, a standard and minimally invasive procedure in elasmobranch bioluminescence research.^25^^,^^30^^,^^58^ As animals were obtained opportunistically as fisheries bycatch—a situation in which the species, number, and condition of individuals cannot be anticipated—prior ethics committee approval was not practicable; this is particularly relevant for a deep-sea species such as *D. licha* that is exceptionally rarely encountered alive and cannot be maintained in captivity. Following completion of all measurements, individuals were euthanized by full transection of the spinal cord at the level of the first vertebra, following standard humane killing procedures for elasmobranchs and in accordance with institutional guidelines. Following euthanasia, animals were retained as voucher specimens or kept for follow-up histological and biochemical analyses.

### Method details

#### Spontaneous luminescence analyses

Of the kitefin sharks obtained as bycatch during the survey, eight individuals exhibiting spontaneous, stable bioluminescence under dark conditions were selected for analysis. These animals were alive and in the best apparent physiological condition at the time of measurement, as assessed by their responsiveness, posture, and the stability of their luminescent output over the observation period. Individuals that did not luminesce or whose luminescence was declining, were excluded for these reasons, which are both methodological and physiological. In our experience, luminescence fades before death in bioluminescent sharks—including the dalatiid *Squaliolus aliae* and the etmopterids *Etmopterus spinax* and *E. schmidti*—and counterilluminating animals more generally maintain a stable output at a given level of illumination.^5^ Restricting our sample to the eight individuals that exhibited stable luminescent output was therefore also a conservative approach, minimizing the risk of including compromised animals without requiring us to determine the cause of absent or declining luminescence in any particular individual. This is discussed further in the Discussion and in the Limitations of the study.

Bioluminescent individuals were photographed in complete darkness using a Sony α7SIII camera (ISO 80,000; 20 mm lens; f/1.6; 5–30 s exposure). For display purposes, uniform brightness and contrast adjustments were applied to the entire image in Adobe Photoshop®. The extent of the photogenic area relative to the total projected body silhouette was quantified using ImageJ (Fiji distribution v.2.14.0/1.54f, National Institutes of Health, Bethesda, MD).^59^

Emission spectra were acquired following Gouveneaux and Mallefet^60^ using a Hamamatsu mini-spectrometer (C10083CA) equipped with an optical fiber (A976201). Background spectra recorded from dark regions surrounding luminous specimens were subtracted from all measurements. Spectral measurements provide peak wavelength rather than absolute spectral irradiance; Gaussian curve fitting (adjusted R^2^ > 0.90) was performed using MagicPlot (MagicPlot Systems, 2013).

Spontaneous ventral bioluminescence was measured directly on the skin using an optical fiber probe (Edmund Optics #39–368; core diameter 0.635 cm; numerical aperture 0.55) coupled to a photon-counting luminometer (Berthold FB12), housed inside a black opaque tube and positioned 1 cm from the ventral surface normal using a rigid spacer. The tube restricted the field of view and minimized stray light. Luminescent output was expressed as photon flux per unit skin surface (Mq s^−1^; 1 Mq = 10^6^ photons), following calibration against a certified 470 nm reference light source (Beta light; Saunders Technology, Hayes, UK).

The angular distribution of luminescence was measured following Claes et al.^25^^,^^30^ using the optical fiber–luminometer assembly described above. Relative light intensity was recorded along a circular arc of 7 cm radius at five transverse body positions: rostral, mandibular, pectoral, ventral, and pelvic. Anterior positions (rostral, mandibular, pectoral) were measured over 180° downward arcs; posterior positions (ventral, pelvic) were measured over 360° to capture emission from the first and second dorsal fins, respectively. For these measurements, individuals were briefly emersed in dorsal recumbency (tonic immobility) for the duration of the measurement (∼2–3 min) following Claes et al.,^25^^,^^30^ then returned immediately to aerated seawater; all individuals recovered normally, confirming their physiological responsiveness throughout.

#### Estimation of counterillumination depth

Because the angular emission profile of individual shark ventral photophores is unknown, and given the high photophore density of *D. licha* which allows the ventral surface to be approximated as a continuous emitting layer,^24^ ventral emission was treated as Lambertian over the downward hemisphere. Under Lambert's cosine law, radiant intensity decreases as cos(θ) relative to the surface normal, such that radiance—radiant intensity per unit projected area—remains constant across all emission angles. Under this approximation, radiance *L* relates to photon emittance *E* as *L* = *E*/π (see Mobley, 1994^61^).

At the macroscopic scale relevant for visual detection, high photophore density allows the ventral surface to be approximated as a continuous emitting layer. In the absence of angular emission measurements, ventral emission was approximated as Lambertian over the downward hemisphere for the purpose of converting measured photon flux into an effective mean radiance.

The emitting surface sampled by the measurement was approximated as the tube aperture:

$$A=\pi {\left(\frac{d}{2}\right)}^{2}$$

where Φ_shark_ is the measured photon flux (photons s^−1^) recorded by the luminometer, A is the aperture area of the measurement tube (m^2^), and *d* is the internal diameter of the tube (minimum 0.79 cm).

Photon emittance:

$$E_{\text{shark}}=\frac{\Phi_{\text{shark}}}{A}$$

Radiance:

$$L_{\text{shark}}=\frac{E_{\text{shark}}}{\pi }=\frac{\Phi_{\text{shark}}}{\pi A}$$

Ambient downwelling radiance was modeled using published absolute radiance values at 200 m depth for clear oceanic waters and a depth-dependent attenuation coefficient (k = 1.638 log_10_ units per 100 m).^62^ Downwelling radiance at depth *z*was expressed as:

$$\log_{10}L_{d}\left(z\right)=\log_{10}L_{d}\left(200\right)-k\frac{\left(z-200\right)}{100}$$

Because the reference radiance is provided for a solar elevation of 45°, downwelling radiance was adjusted using the maximum solar elevation of the capture day (*h*):

$$L_{d}\propto \sin\left(h\right)$$

For each ventral luminescence measurement, counterillumination depth (*z*∗) was defined as the depth at which ventral radiance equaled ambient downwelling radiance:

$$L_{\text{shark}}=L_{d}\left(z^{*}\right)$$

Solving yields:

$$z^{*}=200+\frac{\log_{10}L_{d}\left(200\right)-\log_{10}L_{\text{shark}}}{k}\times 100$$

### Quantification and statistical analysis

All statistical analyses were conducted in Python using the SciPy and statsmodels libraries. Statistical significance was set at α = 0.05. Where asterisks are used to denote statistical significance in the figures (Figure 3), they refer to FDR-corrected *q*-values from the two-sided one-sample Student's *t*-tests described below (∗*q* < 0.05, ∗∗*q* < 0.01, ∗∗∗*q* < 0.001), as defined in the corresponding figure legend.

Differences among anatomical zones were tested using a repeated-measures analysis of variance (ANOVA) with zone as a within-subject factor and individual as subject. Model assumptions were assessed using residual diagnostics, Shapiro–Wilk tests for normality, and Levene’s tests for homogeneity of variance. Both assumptions were satisfied (Shapiro–Wilk, *p* = 0.25; Levene, *p* = 0.10), supporting the use of parametric approaches.

To provide a non-parametric confirmation independent of distributional assumptions, a Friedman test was additionally performed.

Differences between counterillumination and capture depth were evaluated using a paired Student’s *t* test, with condition treated as a within-subject factor and each paired observation corresponding to the same sampling unit. Model assumptions were assessed using inspection of residuals and a Shapiro–Wilk test on paired differences to evaluate normality. The normality assumption was satisfied (Shapiro–Wilk, W = 0.904, *p* = 0.317), supporting the use of a parametric paired analysis. To provide a non-parametric confirmation independent of distributional assumptions, a Wilcoxon signed-rank test was additionally performed.

For each anatomical position, the normalized angular emission profile of *D. licha* specimens (*n* = 8) was compared with the angular distribution of residual downwelling light, approximated by the theoretical Tyler distribution.^11^ Comparisons were performed on an angle-by-angle basis using two-sided one-sample *t*-tests. To account for multiple comparisons across all angles and anatomical positions, false discovery rate correction was applied.

Angular emission profiles were compiled for all counterilluminating taxa considered, including sharks, teleost fishes, and crustaceans reported in the literature (*Argyropelecus affinis*,^11^ *Chauliodus sloani*,^11^ *E*. *spinax*,^31^ *E*. *splendidus*,^25^ *Leiognathus equula*,^63^ *Porichthys notatus*,^20^ *Sergestes similis*,^7^ *Squaliolus aliae*^25^), together with data from *Etmopterus joungi*, *E*. *lucifer* and *E*. *schmidti* (Table S3; Claes and Mallefet, unpublished data).

Angular radiance profiles were integrated over the downwelling hemisphere into three angular sectors (downward, oblique, lateral). Sector boundaries were defined using a residual downwelling-light model such that the model contributed equally (1/3) to each sector, ensuring that residual downwelling light occupied the center of ternary space. For each genus, an illustrative outline was drawn based on fresh specimens morphology using Powerpoint (Microsoft Office suite) and mapped onto the plot for illustrative purpose.

Sector fractions were treated as compositional data and analyzed in Aitchison geometry^64^ following isometric log-ratio (ILR) transformation.^65^ Where replication allowed, species-level mean compositions were calculated prior to analysis. Similarity to residual downwelling light was quantified using Aitchison distance.

Statistical comparisons were performed on ILR-transformed coordinates using permutation-based tests (10,000 permutations), based on differences between group centroids in ILR space. Because replication within *Etmopterus* genus was limited, deviation of *Etmopterus* emission profiles from residual downwelling light (ILR = 0) was assessed using a one-sample sign-flip permutation test on ILR-transformed compositions.

## Supplemental information

Document S1. Tables S1 and S3 and S4

Table S2. Downward (ventral photogenic areas) angular emission data for *Dalatias licha*, related to Figures 2 and 3 (A) Mandibular region. (B) Rostral region. (C) Pectoral region. (D) Ventral region. (E) Pelvic region
