# Supplementary material for: Counterillumination in a large demersal bioluminescent shark
Source: iScience. 2026 Aug 6;29(8):117089. doi: 10.1016/j.isci.2026.117089 (PMC13475669; doi:10.1016/j.isci.2026.117089)
Supplement: Supplementary file 1 [file mmc1.pdf]

iScience, Volume *29*

Supplemental information

Counterillumination in a large demersal bioluminescent shark

Julien M. Claes, Darren W. Stevens, and Jérôme Mallefet

Table S1. Specimen information for the 8 luminescent individuals included in the analysis. Related to Results.

Shark #	Sex	Total length (cm)	Capture depth min (m)	Capture depth max (m)	Latitude (°)	Longitude (°)	Capture date
1	Male	58	533	536	42.95517 S	178.73567 W	2024-01-12
2	Male	68.5	517	522	43.18833 S	179.96767 W	2024-01-10
3	Male	49.5	487	491	44.09650 S	178.00883 W	2024-01-18
4	Male	54.5	487	491	44.09650 S	178.00883 W	2024-01-18
5	Female	85	487	491	44.09650 S	178.00883 W	2024-01-18
6	Male	54.5	649	657	42.86667 S	175.93050 E	2024-01-29
7	Male	64	649	657	42.86667 S	175.93050 E	2024-01-29
8	Male	39	554	562	42.96567 S	175.75217 E	2024-01-31

Table S3. Upward (dorsal fins) angular emission data for *Dalatias licha*. Related to Figure 2.

	Fin 1 (% of ventral emission)								Fin 2 (% of ventral emission)						
	**Shark 1**	**Shark 2**	**Shark 3**	**Shark 4**	**Shark 5**	**Shark 6**	**Shark 7**	**Shark 8**	**Shark 1**	**Shark 2**	**Shark 3**	**Shark 4**	**Shark 5**	**Shark 8**	**Tyler****
-90	0.0556	0.0742	0.046	0.2164	0.1959	0.0774	0.2526	0.0655	0.1116	0.1451	0.1549	0.3839	0.2241	0.1591	0.0002
-80	0.0642	0.0774	0.0553	0.1984	0.1818	0.1148	0.2568	0.0732	0.1195	0.1507	0.1439	0.3896	0.2478	0.1684	0.0003
-70	0.0377	0.0788	0.0583	0.1962	0.1751	0.153	0.2558	0.075	0.1215	0.148	0.1199	0.3758	0.2384	0.162	0.0004
-60	0.0407	0.0894	0.0548	0.1761	0.1735	0.2005	0.2436	0.0755	0.1191	0.1333	0.1133	0.3393	0.2781	0.1866	0.0006
-50	NA	0.0923	0.0551	0.1535	0.1574	0.2396	0.2312	0.0696	0.1016	0.1256	0.0995	0.2953	0.2656	0.1622	0.0009
-40	0.0321	0.095	0.0509	0.1431	0.1383	0.3008	0.2094	0.0613	0.078	0.1107	0.089	0.2757	0.0267	0.1586	0.0015
-30	NA	0.0923	0.0485	0.1431	0.1161	0.3375	0.1782	0.05	0.0528	0.1018	0.075	0.2404	0.2206	0.1693	0.0023
-20	0.0407	0.095	0.0475	0.141	0.0958	0.3804	0.1634	0.047	NA	0.1047	0.063	0.2079	0.177	0.148	0.0034
-10	0.0321	0.0929	0.04	0.1306	0.0794	0.4006	0.1356	0.042	0.0461	0.1005	0.053	0.1879	0.1134	0.1237	0.0045
0	0.0597	0.0791	0.04	0.1205	0.0695	0.4336	0.1272	0.0389	0.0784	0.0972	0.0513	0.1753	0.1035	0.1193	0.005
10	0.0321	0.0929	0.04	0.1306	0.0794	0.4006	0.1356	0.042	0.0461	0.1005	0.053	0.1879	0.1134	0.1237	0.0045
20	0.0407	0.095	0.0475	0.141	0.0958	0.3804	0.1634	0.047	NA	0.1047	0.063	0.2079	0.177	0.148	0.0034
30	NA	0.0923	0.0485	0.1431	0.1161	0.3375	0.1782	0.05	0.0528	0.1018	0.075	0.2404	0.2206	0.1693	0.0023
40	0.0321	0.095	0.0509	0.1431	0.1383	0.3008	0.2094	0.0613	0.078	0.1107	0.089	0.2757	0.0267	0.1586	0.0015
50	NA	0.0923	0.0551	0.1535	0.1574	0.2396	0.2312	0.0696	0.1016	0.1256	0.0995	0.2953	0.2656	0.1622	0.0009
60	0.0407	0.0894	0.0548	0.1761	0.1735	0.2005	0.2436	0.0755	0.1191	0.1333	0.1133	0.3393	0.2781	0.1866	0.0006
70	0.0377	0.0788	0.0583	0.1962	0.1751	0.153	0.2558	0.075	0.1215	0.148	0.1199	0.3758	0.2384	0.162	0.0004
80	0.0642	0.0774	0.0553	0.1984	0.1818	0.1148	0.2568	0.0732	0.1195	0.1507	0.1439	0.3896	0.2478	0.1684	0.0003
90	0.0556	0.0742	0.046	0.2164	0.1959	0.0774	0.2526	0.0655	0.1116	0.1451	0.1549	0.3839	0.2241	0.1591	0.0002

*Zenith

**These relative values correspond to 1/200 of Tyler's prediction, following reference 13.

Table S4. Angular emission data for *Etmopterus* spp. Related to Figure 3.

Angle (°)	*E. joungi*	*E. lucifer*	*E. schmidti* 1	*E. schmidti* 2	*E. spinax* 1*	*E. spinax* 2*	*E. spinax* 3*
0	100	100	100	100	100	100	100
10	70.0271	90.5188	85.0829	99.7535	87.6481	87.2258	89.3071
20	62.1412	82.5614	78.3686	87.1851	66.8606	68.5009	69.6348
30	47.2847	69.6623	57.6975	68.4924	45.6393	42.7523	50.068
40	33.3471	55.6156	41.0322	44.1615	26.9014	23.1546	28.1309
50	23.7553	41.6116	24.539	26.3802	14.4788	12.5083	15.6139
60	17.3859	29.3891	16.2177	17.4402	8.1306	6.9277	9.2163
70	11.9365	20.7003	9.5723	13.6914	3.2123	3.9163	4.3661
80	9.7859	13.9611	8.3327	8.7032	1.6842	2.1627	1.8046
90	5.8824	9.9168	7.5981	7.4066	0.8511	1.4923	1.3142

* The mean value for these three specimens has been published in reference 50.
